# REMEDIATION: Can Transgenic Plants Root Out Pollutants?

**DOI:** 10.1289/ehp.119-a206a

**Published:** 2011-05

**Authors:** Carol Potera

**Affiliations:** **Carol Potera**, based in Montana, has written for *EHP* since 1996. She also writes for *Microbe*, *Genetic Engineering News*, and the *American Journal of Nursing*

A team of researchers at Colorado State University in Fort Collins and Duke University in Durham, North Carolina, have created transgenic plants that turn from green to white when exposed to the explosive 2,4,6-trinitrotoluene (TNT).^1^ Ultimately, the researchers hope, several simple and affordable plants will be developed that can quickly sense a variety of biological and chemical agents.

The research team combined genes from bacteria and plants to construct a modular “de-greening” gene circuit that breaks down chlorophyll, the green pigment in plants, while simultaneously preventing chlorophyll biosynthesis. The gene circuit contains a customized receptor for a specific hazardous agent—in this case, TNT. When the receptor binds its target, it triggers the de-greening reaction.^1^ “The modular circuit can be inserted into any plant,” says study leader June Medford, a biology professor at Colorado State University.

In the new study, transgenic Arabidopsis and tobacco plants turned white when they contacted airborne TNT or when exposed to soilborne TNT through their roots. Second-generation plants grown from seeds of the first plants inherited the ability to sense and respond to TNT.^1^ The color conversion in the laboratory plants took 2 to 3 hours. Medford’s team is working to reduce the response time to minutes rather than hours.

Just picomolar (ppt) or nanomolar (ppb) levels of TNT activated the de-greening process, suggesting the system is feasible for real-world applications.^1^ Dogs trained to sniff out explosives and drugs generally discern these agents at ppb or ppt concentrations.^2^,^3^

The plant technology could be deployed “along travel routes to detect for improvised explosive devices or on training ranges to monitor for TNT contamination in soil or runoff,” says Linda Chrisey, biotechnology program manager at the Office of Naval Research in Arlington, Virginia. According to the Agency for Toxic Substances and Disease Registry, TNT contamination is found on at least 20 National Priorities List sites identified by the U.S. Environmental Protection Agency.^4^ People can be exposed to TNT through eating, drinking, touching, or inhaling contaminated soil, water, food, or air, with potential health effects including anemia, abnormal liver function, skin irritation, and cataracts.^4^

Transgenic sentinel plants should not face disposal concerns, because they do not become chemically saturated, according to Medford. “This is not phytoremediation; we’re talking about exceeding low levels of contaminants,” she says. Moreover, she says, “We don’t intend to put this technology into plants that people eat.”

“This is a revolutionary approach to working with plants as environmental sentinels that looks to have broad application,” says Bill Farland, senior vice president for research at Colorado State University. For instance, plants may someday sniff out air or water pollutants released from industrial sources such as chemical manufacturing plants. Other potential applications, Chrisey notes, include the detection of herbicides on crops, pathogens in municipal water supplies, or explosives in airports.

“We still have to explore all the possibilities of chemicals that could be sensed by plants,” Farland says. The key lies in designing a receptor for a pollutant of choice and engineering it into the plant de-greening circuit. Medford’s team also is designing plants with multiple receptors to detect more than one pollutant.

“Living organisms have advantages as sentinels of pollution,” says Paul Johnson, a professor of physics and astronomy at the University of Wyoming in Laramie. In 2009 he and colleagues in France reported they had genetically engineered tadpoles to detect zinc in water with a portable, flow-through system.^5^ Like Medford’s plants, the transgenic tadpoles carry a receptor for a selected pollutant that triggers fluorescence in about an hour. Also, tadpoles are being designed to detect more than one environmental pollutant—potentially to include heavy metals, organochlorine pesticides, bisphenol A, polychlorinated biphenyls, and dioxins—and to generate several fluorescent colors.^6^

Plants and tadpoles provide relatively cheap monitoring systems, Johnson says, and they give results rapidly onsite, compared with carrying samples back to a laboratory for expensive analysis such as mass spectrometry. Plus, he says, living systems, particularly animal-based ones, reflect physiologic effects of environmental pollutants similar to those that occur in humans. “The detection of contaminants and pollutants in air and water is a rapidly expanding area, and new developments will stay fruitful for a long time,” Johnson says.

## Figures and Tables

**Figure:**
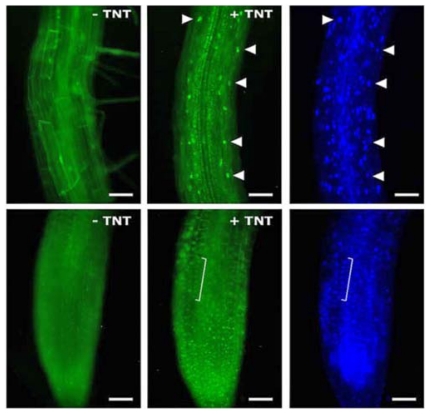
Epifluorescence images of different portions of transgenic *Arabidopsis* roots shown before and after addition of the TNT ligand. Far right panels show DAPI nuclear staining. Arrowheads indicate nuclei. Scale bar = 25 μm.
